# Organization of Rehabilitation Services in Randomized Controlled Trials: Which Factors Influence Functional Outcome? A Systematic Review

**DOI:** 10.1016/j.arrct.2022.100197

**Published:** 2022-04-13

**Authors:** Cecilie Røe, Erik Bautz-Holter, Nada Andelic, Helene Lundgaard Søberg, Boya Nugraha, Christoph Gutenbrunner, Andrea Boekel, Marit Kirkevold, Grace Engen, Juan Lu

**Affiliations:** a Research Centre for Habilitation and Rehabilitation Models and Services (CHARM), Institute of Health and Society, Faculty of Medicine, University of Oslo, Oslo, Norway; b Department of Physical Medicine and Rehabilitation, Oslo University Hospital, Oslo, Norway; c Institute of Clinical Medicine, Faculty of Medicine, University of Oslo, Oslo, Norway; d Department of Physiotherapy, Faculty of Health Sciences, Oslo Metropolitan University, Oslo, Norway; e Department of Rehabilitation Medicine, Hannover Medical School, Hanover, Germany; f Institute of Nursing and Health Promotion, Faculty of Health Sciences, OsloMet University, Oslo, Norway; g Department of Family Medicine and Population Health, Division of Epidemiology, Virginia Commonwealth University, Richmond, Virginia

**Keywords:** Rehabilitation, ICF, International Classification of Functioning, Disability, and Health, ICSO-R, International Classification System for Service Organization in Health-related Rehabilitation, RCT, randomized controlled trial

## Abstract

**Objective:**

To identify factors related to the organization of rehabilitation services that may influence patients’ functional outcome and make recommendations for categories to be used in the reporting of rehabilitation interventions.

**Data Sources:**

A systematic review based on a search in MEDLINE indexed journals (MEDLINE [OVID], Cumulative Index of Nursing and Allied Health Literature, PsycINFO, Cochrane Central Register of Controlled Trials) until June 2019.

**Study Selection:**

In total 8587 candidate randomized controlled trials reporting on organizational factors of multidisciplinary rehabilitation interventions and their associations with functional outcome. An additional 1534 trials were identified from June 2019 to March 2021. **Data Extraction:** Quality evaluation was conducted by 2 independent researchers. The organizational factors were classified according to the International Classification for Service Organization in Health-related Rehabilitation 2.0.

**Data Synthesis:**

In total 80 articles fulfilled the inclusion criteria. There was a great heterogeneity in the terminology and reporting of service organization across all studies. Aspects of Settings including the Mode of Service Delivery was the most explicitly analyzed organizational category (44 studies). The importance of the integration of rehabilitation in the inpatient services was supported. Furthermore, several studies documented a lack of difference in outcome between outpatient vs inpatient service delivery. Patient Centeredness, Integration of Care, and Time and Intensity factors were also analyzed, but heterogeneity of interventions in these studies prohibited aggregation of results.

**Conclusions:**

Settings and in particular the way the services were delivered to the users influenced functional outcome. Hence, it should be compulsory to include a standardized reporting of aspects of service delivery in clinical trials. We would also advise further standardization in the description of organizational factors in rehabilitation interventions to build knowledge of effective service organization.

Effective organization of rehabilitation services integrating the medical perspectives with vocational, educational, and community support are necessary to meet the complex challenges facing the field of rehabilitation. The term service is derived from the act of serving and refers to the provision of intangible products, and rehabilitation services refers to the provision of intangible products to maintain or improve functioning.^1^ Organization of the services refers to purposefully designed, structured social system developed for the delivery of health care services and comprises provision and delivery of the services.^1^

The services can be viewed from diverse perspectives, including from societal, institutional, and individual levels. These levels are often referred to respectively as macrolevel, including the policy and financial aspects; mesolevel, including organization and availability of the services; and microlevel, including the accessibility and content of services provided to the individual patient.^2^ Donabedian^3^ described the quality of the services as the causal relationship between the attributes of setting and the process of care and linked them to the outcome. Evaluating the quality of rehabilitation services is important on every level, but the challenges of evaluation may increase when moving from the micro- to the meso- and macrolevels.

A wide variety of rehabilitation interventions have been developed for the different functional problems caused by diseases or trauma. Greater knowledge about how rehabilitation interventions should be implemented in the services is needed to maximize functional outcomes.^4^ In studies testing rehabilitation interventions, the description of different aspects of service provision and delivery often lacks systematic approaches.^5^ Hence, organizational factors may not be included in the analyses even though a recent systematic review has suggested that these factors could have significant effect on the outcome.^6^ The lack of a framework for depicting differences in service delivery may contribute to the knowledge gap regarding optimal rehabilitation service delivery.

Gutenbrunner et al^7^ proposed a classification for organization of rehabilitation services, the International Classification System for Service Organization in Health-related Rehabilitation (ICSO-R), describing the mesolevel of services. A revised version ICSO-R 2.0 has also been published recently.^8^ It includes 2 dimensions of Service Provider and Service Delivery, 21 specified categories, and 17 subcategories (table 1). The Service Provider dimension describes where, by whom, and in which context the services are delivered. For example rehabilitation services at Oslo University Hospital would refer to Oslo (where/location), public (whom/organization) because the services in Norway are public, Hospital (Context). The Service Delivery dimension describes the characteristics of the interventions, procedures, and users of the services. For example, for early rehabilitation delivered to individuals with traumatic brain injuries, traumatic brain injuries refers to the category Target group and acute phase/early rehabilitation to the category Aspects of Time.

**Table 1:** International Classification of Service Organization in Rehabilitation (ICSO-R 2.0) with its 21 specified categories

Provider	Service Delivery
1.1 Context	2.1 Health Strategies
1.2 Ownership	2.2. Service Goals
1.3 Location	2.3 Target Groups (subcategories 2.3.1 Health Condition, 2.3.2 Functioning, 2.3.3 Other Target Groups)
1.4 Governance/leadership (subcategories 1.4.1 Mission, 1.4.2 Vision, 1.4.3 Involvement in Governance and Management)	2.4 Modes of Referral
1.5 Quality Assurance and Management	2.5 Location of Service Delivery (subcategories 2.5.1 Location Characteristics, 2.5.2 Catchment area)
1.6 Human Resources	2.6 Facility
1.7 Technical Resources	2.7 Setting (subcategories 2.7.1 Levels of Care, 2.7.2 Mode of Service Delivery and 2.7.3 Phase of Health Care)
1.8 Funding of service provider (subcategories 1.8.1 Source of money, 1.8.2 Criteria of Spending)	2.8 Integration of Care
	2.9 Patient-Centeredness
	2.10 Aspects of Time and Intensity
	2.11 Rehabilitation Team (subcategories 2.11.1 Profession, Competencies, 2.11.2 Interaction Approaches
	2.12 Reporting and Documentation
	2.13 Funding of Service Delivery (subcategories 2.13.1 Source of Money, 2.13.2 Criteria of Payment)

NOTE. ICSO-R 2.0 also comprises 17 subcategories.

The effect of a rehabilitation intervention is well known to be influenced by personal and contextual factors. Personal factors are presently reflected in the Consolidated Standards of Reporting Trials guidelines.^9^ Accordingly, age and sex of the participants in rehabilitation trials are routinely reported. Aspects of Organization of the services may be equally relevant in rehabilitation^10^ but is seldom reported in trials. Hence, better understanding of the interaction between service delivery at the mesolevel and the content of the rehabilitation at the microlevel is fundamental. Short but systematic description and analytical approach to the service factors are also needed in rehabilitation trials when their main interventional focus is at the microlevel. The implementation of the International Classification of Functioning, Disability, and Health (ICF)^11^ has provided the advantage of having a common language across disciplines and countries. The application of reporting guidelines developed for randomized controlled trials (RCTs), such as the guidelines,^9^ has facilitated standardized reporting of RCTs and meta-analysis of important factors influencing intervention effects at the microlevel.^12^

However, a review of recent randomized rehabilitation trials suggested that the service provider and delivery as assessed by categories in the ICSO-R 2.0 varied widely and recommended standardizing the descriptions of services in future RCTs.^5^ Further, the list of ICSO-R 2.0 categories is too long to fit into the reporting format for clinical trials. Hence, a minimum reporting set for service organization characteristics is needed and should contain factors documented to influence outcome of clinical trials.

The development of ICSO-R is theory-driven^1^ and based on the biopsychosocial model that asserts that the rehabilitation actions are closely linked to functional outcome.^13^ Yet, to our knowledge, no systematic overview of scientific evidence has been undertaken regarding the effect of the ICSO-R recommended categories for service provision on the patient outcomes. Thus, the aim of the present study is to identify factors of provision and delivery of rehabilitation services (mesolevel) that may influence patients’ functional outcome (microlevel) and recommend categories of service provision and delivery that should be included in the reporting of rehabilitation RCTs.

## Methods

This review was carried out in accordance to the Preferred Reporting Items for Systematic Reviews and Meta-Analyses guidelines,^14^ and the study protocol was registered in the International Prospective Register of Systematic Reviews 21.10.19, registration number CRD42020151832.

### Systematic review search strategy

MEDLINE indexed journals (MEDLINE [OVID], Cumulative Index of Nursing and Allied Health Literature, PsycINFO, Cochrane Central Register of Controlled Trials) were searched between database inception and June 2019 for all RCTs that provide rehabilitation intervention and updated March 2021 (appendix S1).

### Study inclusion criteria

The inclusion criteria for articles were elaborated according to the Problem, Intervention, Comparison, Outcomes framework^15^ and targeted to disease or trauma that has the potential to cause long-term disabilities. All RCTs including a rehabilitation intervention in at least 1 of the intervention arms were eligible for the current review. Rehabilitation interventions were operationalized as interventions delivered by 2 or more health professions aimed at improving patients’ functioning. Each intervention arm should include 10 or more participants, and the categories and subcategories of Service provision and delivery as described by ICSO-R 2.0 (see table 1) should be described and analyzed. Finally, the studies were included if they addressed health and functioning in the ICF perspective (body functions, activities, participations) or health-related quality of life in the outcome evaluation. RCT reports not in English were excluded.

### Article selection

**Fig 1:**
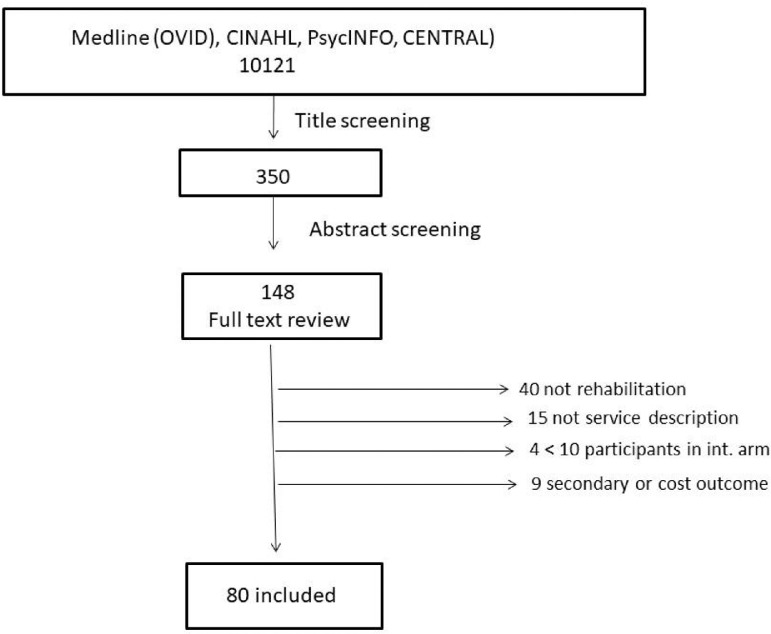
Flow chart of inclusion process. Abbreviations: CINAHL, Cumulative Index of Nursing and Allied Health Literature; CENTRAL, Cochrane Central Register of Controlled Trials.

A total of 8587 articles were originally identified (until June2019), and 1534 were added in the updated search (March 2021) and imported to EndNote. One researcher screened the titles and abstracts and identified 148 studies for full-text review. Two researchers independently screened the full texts regarding the fulfillment of the inclusion criteria. After the full-text evaluation, 40 studies were excluded because of the absence of rehabilitation interventions or lack of service descriptions in 1 or more of the intervention arms.^15^ Four studies were excluded because of insufficient participant numbers (<10) in 1 of the intervention arms. Nine articles reported the cost effectiveness or the secondary outcome of studies that already had been included and were thus excluded. Hence, 80 articles were included in current review (fig 1).

### Data extraction

A protocol was developed based on the definitions of the categories and subcategories of ICSO-R 2.0 to guide and standardize the data extraction process. Data extraction was performed independently by 2 authors (C.R., E.B.H.). The data included primary authors and the study's publication year, targeted groups, types of intervention and settings, sample size in each intervention arm that contributed to the study outcome analysis, and functional and/or quality of life outcomes. In studies with multiple outcomes, generic functioning or quality of life measurements was chosen as the main outcome to address the objective of the current review. When several follow-up points were reported, the outcome measurement at the last follow-up time point was used to assess differences in outcome. Two authors (C.R., E.B.H.) categorized the main differences in service organization between the intervention arms according to ICSO-R 2.0 categories and subcategories. The categorization was based on the stated aim of the original study, the description of the intervention arms, and the factors addressed in the analyses of the respective studies. An interactive consensus-based approach for classification of the studies according to the 1 main ICSO-R 2.0 category differentiating the intervention arms in the studies was adopted. Well-described and important covarying categories are described and reported in the summary tables.

### Methodological quality and risk of bias assessment

Quality evaluation of each eligible study was performed independently by 2 researchers (C.R., E.B.H.) according to the 16 quality items suggested by Cicerone et al.^16^ The 16 items include 8 items of internal validity, 6 items of study description, and 4 items of statistical quality (supplemental S2). For each item, satisfactory assessment scores 1 point, and the highest quality score for each study consists of 16 points. Discrepancy between the raters occurred in 29 items overall (2.3%). Disagreement was resolved through consensus before reaching a total score. The scores are reported for all studies.

### Data analyses and synthesis

Data were synthesized descriptively and presented according to the nature of the interventions and rehabilitation settings (C.R., J.L.), along with the primary authors and publication years, quality assessment results, targeted patient groups, intervention arms, and functional and/or quality of life outcomes. The interventions were also described according to whether they took place in or outside hospitals and in the community or at home. Interventions taking place in the community or in the users’ home with support from hospital-based and/or specialized services staff were denoted as outreach. The outcomes were reported by intervention arms and as a difference between the arms. For the studies that did not report outcome differences, we simply calculated an absolute outcome difference between the study arms based on the original report and presented the differences with an annotation in the summery tables. The procedure was not corrected for risk of bias. Because of wide variations in the outcomes reported and analyzed among studies, the current study is only able to present plots without aggregating effect size based on a limited number of studies that had comparable interventions and outcomes according to the ICSO-R 2.0 categories.

## Results

The quality rating of the 80 included studies, calculated according to the recommendations by Cicerone et al,^16^ varied from 8-15, with a mean score of 12.43±1.80. The studies varied according to the target group, organizational factors analyzed, and outcome evaluated (tables 2-7, supplemental S3). The most frequent condition studied was stroke (25 studies, 30%). Differences in Setting (ICSO-R 2.0 category 2.7) were reported in 44 studies (55%), all generally touching on the Mode of Service Delivery (ICSO-R 2.0 subcategory 2.7.2), that is, the way services are delivered to the users (tables 2-4). In 6 studies Integration of Care (ICSO-R 2.0 category 2.8) and in 4 studies Patient Centeredness (ICSO-R 2.0 category 2.9) were clearly evaluated in the comparison of intervention arms (see tables 5 and 6). In 14 studies, Aspects of Time and Intensity (ISCO-R 2.0 category 2.10) were discussed, and these aspects varied between the intervention arms. In the remaining 12 studies, the organizational differences between the interventions were difficult to categorize according to ICSO-R 2.0, or the control groups received variable treatment (see supplemental S3).

**Table 2:** Setting differences in inpatient rehabilitation (ICSO-R 2.0 category 2.7). Target group, quality score according to Cicerone 2009, brief mapping of the intervention arms with number of randomized subjects, and the outcome (generic functioning or quality of life measurement reported when multiple outcomes). Group difference, with effect size and statistical level reported when possible.

Studies	Quality Rating (Mean)	Target Group	Inpatient Rehabilitation 1	Inpatient Rehabilitation 2	Functional Measures Outcome Difference (Inpatient Rehabilitation 1 vs 2)
**2.7 Setting** (main subcategory differentiating the intervention arms)					
Munin et al. 2005^17^ (2.7.2 Mode of service delivery)	10	Elderly hip fractures	Rehabilitation facility (n=42) Mean: 31	Skilled nurse facility (n=34) Mean: 21	FIMmotor 12 wk after discharge Difference in mean: 10*, *P*=.034
Kalra and Eade 1995^40^ (2.7.2 Mode of service delivery)	11	Stroke	Stroke unit (n=34) Change in median: 5	General ward (n=37) Change in median: 3	Barthel Index at discharge† Difference in median change: 2*, NS
Kalra 1994^41^ (2.7.2 Mode of service delivery)	10	Stroke	Stroke unit (n=73) Median: 15 (Range 6-20)	General ward (n=68) Median: 12 (Range 2-18)	Barthel Index at discharge Difference in median: 3*, *P*=.001
Kalra et al. 1993^42^ (2.7.2 Mode of service delivery)	10	Stroke	Stroke unit (n=75) Median: 15 Change in median: 12	General ward (n=71) Median: 13 Change in median: 8	Barthel Index at discharge‡ Difference in median: 2*, *P*<.05 Difference in median change: 4*, *P*<.05

Abbreviation: NS, no significant group difference.

⁎ Outcome difference was calculated by current study descriptively (for example, 12119 Munin et al., difference in mean=31-21=10).

† Data reported here was from the year of 1994 in which the study design was relevant to the current review.

‡ The original analysis on Barthel Index was stratified by the prognostic scores (<3, 3-5, >5). The results reported here were from patients with the score of 3-5; there was no deference in the Index for patients with the score of <3 or >5.

### Mode of Service Delivery (subcategory 2.7.2 in ICSO-R 2.0)

#### Comparison of different inpatient settings

**Fig 2:**
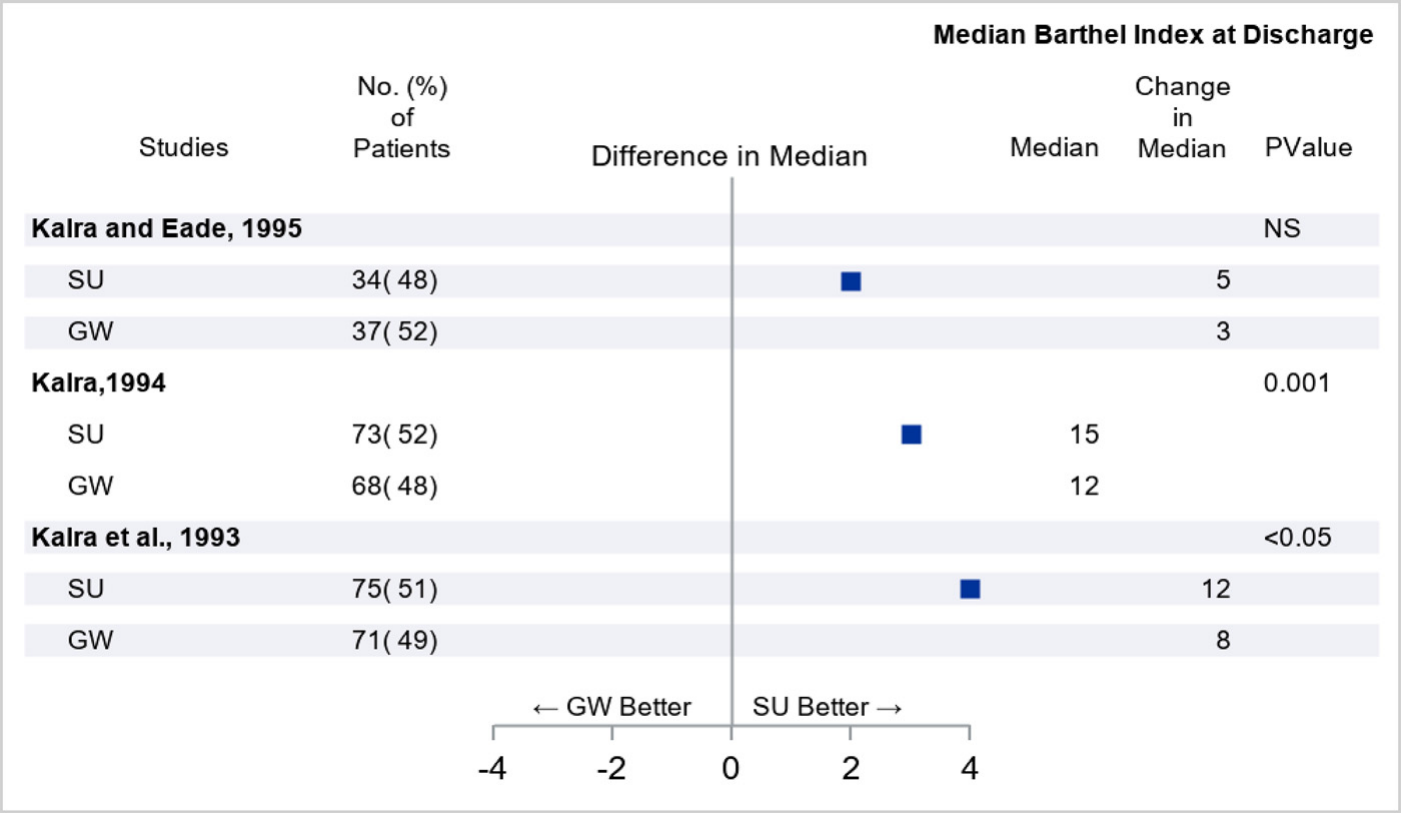
Comparison between different inpatient rehabilitation settings, eg, SU with GW regarding functional outcome as measured by Barthel Index. The targeted groups were patients with stroke. Abbreviations: GW, general ward; SU, stroke unit.

Four studies analyzed the effect of integrated medical and rehabilitation in hospital wards with skilled nursing facilities in a total of 434 patients (see table 2). The study by Munin et al^17^ found significantly larger improvement in functioning, as measured by FIM, when the elderly patients with hip fractures were treated in an integrated rehabilitation facility compared with those in a skilled nursing facility. The other 3 studies (fig 2, see table 2) included patients with stroke and favored integrated rehabilitation in stroke units over control conditions with differences in outcome being statistically significant in 2 of the studies.

#### Inpatient vs outpatient service delivery

**Fig 3:**
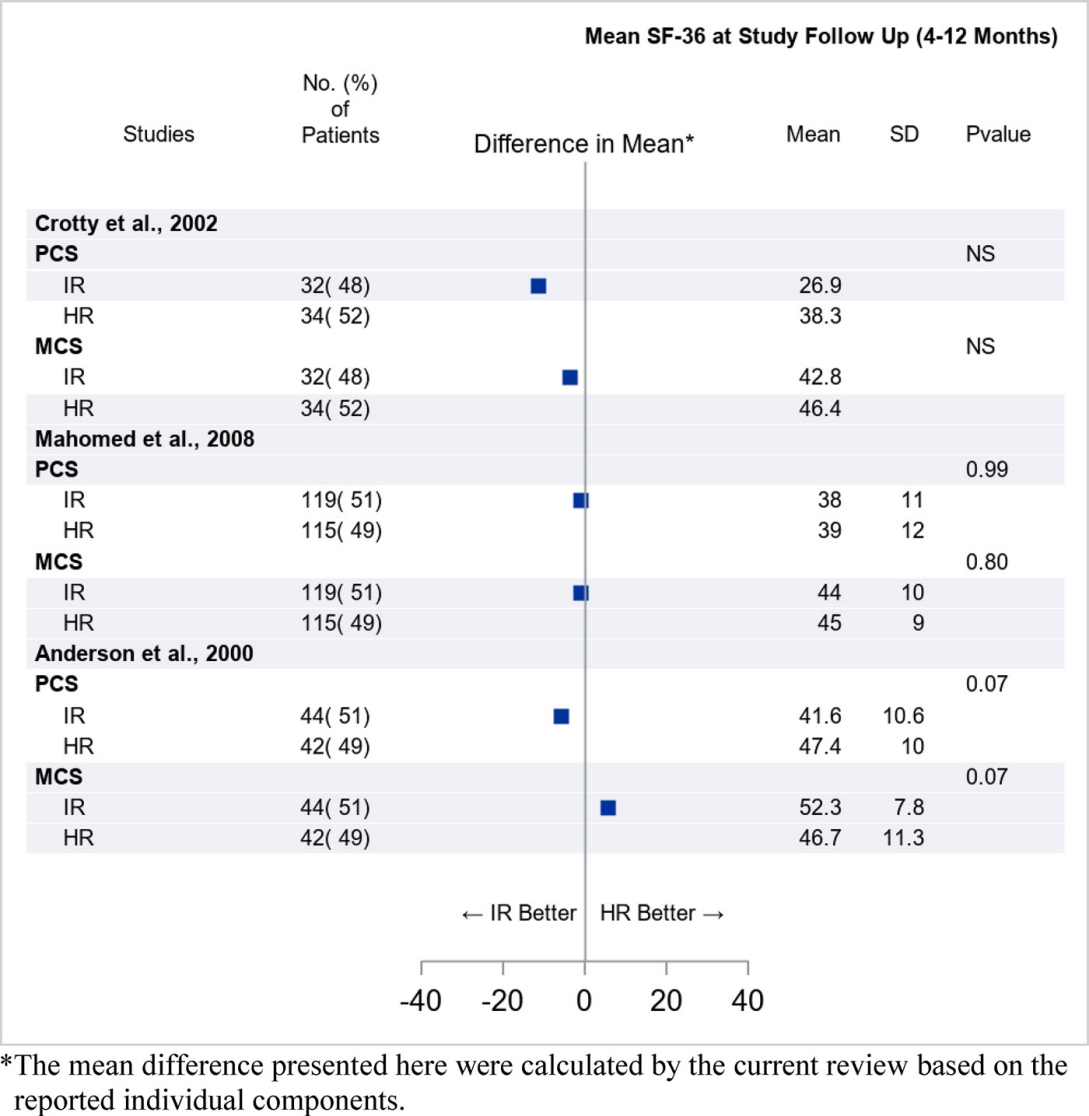
Comparison between IR and HR settings on quality of life outcome among studies reported by the Short Form-36 Health Survey at various lengths of follow-up. The targeted groups were patients on cardiac rehabilitation, elderly patients with hip fractures, and patients with osteoarthritis of the hip and knee, or stroke. Abbreviations: HR, home-based rehabilitation; IR, inpatient rehabilitation; MCS, Mental Component Summary; PCS, Physical Component Summary.

The effects of inpatient settings vs different outpatient settings were compared in 14 studies comprising a total of 2598 patients (see table 3). In 5 of the 14 studies, the quality of life outcome was evaluated with Short Form-36 Health Survey (fig 3). Mutwalli et al^18^ indicated improved outcome in patients with cardiac condition in the outpatient compared with inpatient rehabilitation setting. However, the nonsignificant differences in the other 4 studies are equally important, reflecting noninferiority of outpatient rehabilitation in patients with fractures, orthopedic conditions, and stroke (see fig 3, table 3). The remaining 7 studies reported on heterogeneous target groups and functional outcomes (see table 3). Two studies^19^^,^^20^ favored inpatient compared with outpatient rehabilitation. Sigurdsson et al^21^ indicated lower costs of outpatient rehabilitation in patients with osteoarthritis. The other studies did not reveal differences between in- and outpatient setting, including the lack of effect on sickness absence of supplementary workplace intervention added to comprehensive inpatient rehabilitation.^22^

**Table 3:** Setting differences inpatient vs outpatient rehabilitation (ICSO-R 2.0 category 2.7)

Studies	Quality Rating (Mean)	Target Group	Inpatient Rehabilitation	Outpatient Rehabilitation	Quality of Life Measures (SF-36 Score) Difference in Total Mean Score (Inpatient vs Outpatient Rehabilitation)
**2.7 Setting** (main subcategory differentiating the intervention arms)					
Mutwalli et al. 2012^18^ (2.7.2 Mode of service delivery)	12	Cardiac rehabilitation	Hospital rehabilitation (n=21) Mean ± SD: 60.6 ± 16.2	Home-based outreach (n=28) Mean ± SD: 90.1 ± 4.8	Total SF-36 score at 6 mo follow-up Difference in mean: -29.5*, *P*<.001
Crotty et al. 2002^43^ (2.7.2 Mode of service delivery)	13	Hip fracture	Hospital rehabilitation (n=32) Mean PCS (95% CI): 26.9 (10.2-42.0) Mean PCS change: -3.9 (-19.5 to 11.7) Mean MCS (95% CI): 42.8 (31.2-54.4) Mean MCS change: -11.7 (-23.4 to 0.05)	Early discharge, home-based rehabilitation (n=34) Mean PCS (95% CI): 38.3 (27.9-48.7) Mean PCS change: -3.4 (-14.9 to 8.1) Mean MCS (95% CI): 46.4 (36.2-56.6) Mean MCS change: 0.01 (-13.8 to 13.8)	Total SF-36 at 4 mo after randomization Difference in mean PCS: -11.4* Difference in mean PCS change: 0.5* Difference in mean MCS: -3.6* Difference in mean MCS change: 11.7*
Mahomed et al. 2008^44^ (2.7.2 Mode of service delivery, 2.7.1 Levels of care)	12	Osteoarthritis, hip, knee	Hospital rehabilitation (n=119, 51%) Mean PCS ± SD: 38 ± 11 Mean MCS ± SD: 44 ± 10	Home-based rehabilitation (n=115, 49%) Mean PCS ± SD: 39 ± 12 Mean MCS ± SD: 45 ± 9	Total SF-36 at 12 mo after randomization Difference in mean PCS: -1* Difference in mean MCS: -1*
Anderson et al. 2000^45^ (2.7.2 Mode of service delivery)	14	Stroke	Hospital rehabilitation (n=25) Mean PCS ± SD: 41.6 ± 10.6 Mean MCS ± SD: 52.3 ± 7.8	Early discharge, home-based rehabilitation (n=24) Mean PCS ± SD: 47.4 ± 10.0 Mean MCS ± SD: 46.7 ± 11.3	Total SF-36 at 6 mo after randomization Difference in mean PCS: -5.9 Difference in mean MCS: 5.6
Ronning and Guldvog 1998^46^ (2.7.2 Mode of service delivery, 2.7.1 Levels of care)	12	Stroke	Hospital rehabilitation (n=127) Mean PCS: 48 ± 19 Mean MCS: 70 ± 17	Community based rehabilitation (n=124) Mean PCS: 47 ± 20 Mean MCS: 70 ± 19	Total SF-36 at 7 mo after stroke Difference in mean PCS: 1* Difference in mean MCS: 0*
**Functional Measures**					
Ricauda et al. 2004^47^ (2.7.2 Mode of service delivery)	11	Stroke	Hospital rehabilitation (n=36) Median (IQR): 96.5 (56.5-16.5)	Home-based rehabilitation, outreach (n=39) Median (IQR): 106.0 (67.5-121.5)	Total FIM at 6 mo follow-up Difference in median: -9.5*, *P*=.26
Ozdemir et al. 2001^19^ (2.7.2 Mode of service delivery)	11	Stroke	Hospital rehabilitation (n=30) Mean ± SD: 59.6 ± 14.2	Outpatient rehabilitation (n=30) Mean ± SD: 12.3 ± 13.4	Total FIM at 2 mo follow-up Difference in mean: 47.3*, *P*<.001
Kalra et al. 2000^20^ (2.7.2 Mode of service delivery)	14	Stroke	Hospital rehabilitation Stroke unit/General ward (n=152/146) % of favorable score (15-20): 87%/69%	Home-based rehabilitation, outreach (n=151) % of favorable score (15-20): 71%	Barthel Index at 12 mo after stroke Odds ratio (95% CI) S.Unite vs Outreach: 1.22 (1.09-1.37), *P*<.001 Ward vs Outreach: 0.97 (0.85-1.11), *P*=.65
Hofstad et al. 2014^48^ (2.7.2 Mode of service delivery, 2.7.1 Levels of care)	13	Stroke	Hospital rehabilitation (n= 60) % of BI <95: 66.7%	Day hospital, early discharge or home-based rehabilitation (n=153) % of BI <95: 72.5%	Barthel Index at 6 mo follow-up Difference in % of BI <95: -5.8%*, *P*=.395
Mas et al. 2017^49^ (2.7.2 Mode of service delivery)	10	Elderly fragile	Hospital rehabilitation (n=602) Median (IQR): 69.7 (67.1-72.2)	Home hospital (n=244) Median (IQR): 67.9 (64.3-71.6)	Barthel Index at discharge Difference in median: 1.8*, *P*=.036
Scalvini et al. 2013^50^ (2.7.2 Mode of service delivery)	10	Cardiac surgical	Hospital rehabilitation (n=100) Mean ± SD: 354 ± 102	Home-based rehabilitation, telemonitored specialized care (n=100) Mean ± SD: 334 ± 90	6-min walk test after 4 wk rehab Difference in mean: 20*, *P*>.05
Sigurdsson et al. 2008^21^ (2.7.2 Mode of service delivery)	11	Osteoarthritis hip	Hospital rehabilitation (n=23)	Home-based rehabilitation, outreach, (n=27)	Cost evaluation based on Oxford hip score 6 mo after operation Outreach intervention was associated lower cost
Thorsen et al. 2006^51^ (2.7.2 Mode of service delivery)	8	Stroke	Hospital rehabilitation (n=24)	Early discharge, home-based rehabilitation outreach (n=30)	Need for assistance and health services at 5-y follow-up No significant difference
Skagseth et al. 2020^22^ (2.7.2 Mode of service delivery)	14	Mixed	In and outpatient rehabilitation with work place intervention (n=68)	In and outpatient rehabilitation (n=81)	Sickness absence first 12 mo No group difference

NOTE. Target group, quality score according to Cicerone 2009, brief mapping of the intervention arms with number of randomized subjects, and the outcome (generic functioning or quality of life measurement reported when multiple outcomes). Group difference, with effect size and statistical level reported when possible.

Abbreviations: BI, Barthel Index; IQR, interquartile range; MCS, Mental Component Summary; PCS, Physical Component Summary; SF-36, Short Form Health Survey 36.

⁎ Outcome differences were calculated by current study descriptively (for example, 1974 Mutwalli et al. Difference in mean=60.6-90.1=-29.5).

#### Comparison of different outpatient settings

**Fig 4:**
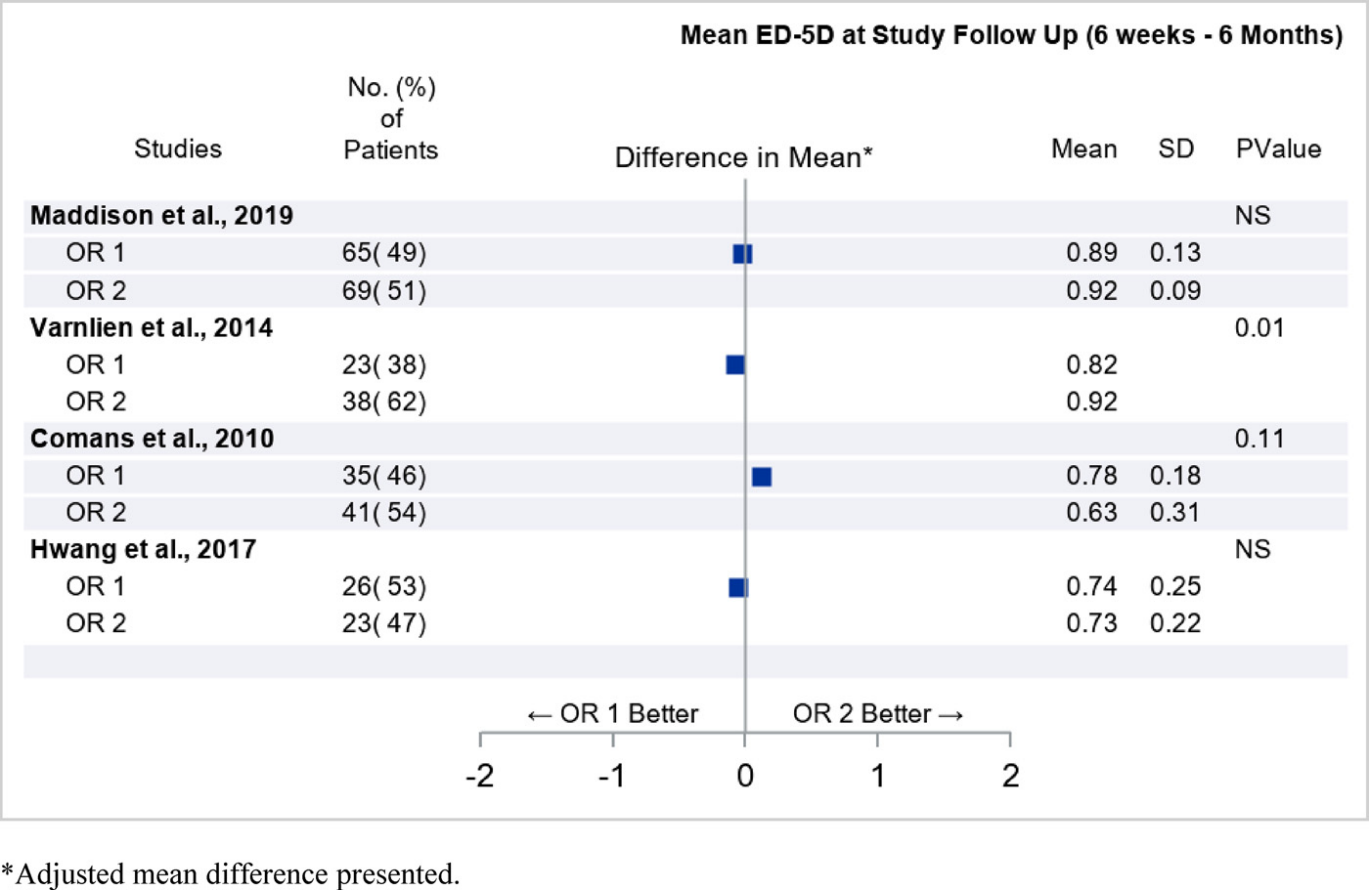
Comparison between different outpatient rehabilitation settings (OR 1 and OR 2) on quality of life evaluated by EuroQol 5D. The targeted groups were patients with different heart conditions or elderly patients. Abbreviations: NS, not significant; OR, outpatient rehabilitation.

A total of 26 studies with 3731 patients evaluated different aspects of settings in outpatient rehabilitation (see table 4). A total of 14 studies that included quality of life as outcome compared outpatient or day based rehabilitation services with home-based or community services. With the exception of 1 study on the intervention for patients with myocardial infarction where the telephone-based home program was found to be superior to outpatient programs,^23^ no differences between the outpatient and home-based or community settings were found (see table 4, fig 4). Among the studies that included functioning as the outcome, the results favored implementation of hearing aids at home, although the effect size was small.^24^ A study among patients with multiple sclerosis revealed greater functional improvement in an outpatient rehabilitation setting than exercising at home,^25^ whereas a patient-centered rehabilitation program provided to veterans with TBI in their homes was superior to outpatient services.^26^

**Table 4:** Setting differences in outpatient service delivery (ICSO-R 2.0 category 2.7)

Studies	Quality Rating (Mean)	Target Group	Outpatient Rehabilitation 1	Outpatient Rehabilitation 2	Outcome Difference Quality of Life Measures (Outpatient Rehabilitation 1 vs 2)
**2.7 Setting** (main subcategory differentiating the intervention arms)					
Kramer et al. 2003^52^ (2.7.2 Mode of service delivery)	12	Knee prothesis	Outpatient rehabilitation (n=69)	Home-based, telephone advices (n=65)	SF-12 12 mo after surgery No significant difference between means*
Oerkild et al. 2011^53^ (2.7.2 Mode of service delivery)	12	Elderly cardiac patients	Outpatient rehabilitation (n=34) PCS and MCS: No difference within group	Home-based rehabilitation outreach (n=30) PCS and MCS: No difference within group	SF-12 at 12 mo follow-up No significant difference between groups*
Mosleh et al. 2015^54^ (2.7.2 Mode of service delivery, 2.7.1.Level of care)	12	Cardiac patients	Outpatient rehabilitation (n=128) Median (IQR) or Mean ± SD on 14 subscales	Home-based rehabilitation (n=109) Same report on 14 subscales	RAND-36 subscales 6 mo after intervention No significant differences between groups
Arthur et al. 2002^23^ (2.7.2 Mode of service delivery)	14	Cardiac	Outpatient rehabilitation (n=109) Mean PCS ± SD: 48.6 ± 7.1	Home-based rehabilitation, Telephone advices (n=113) Mean PCS ± SD: 51.2 ± 6.4	SF-36 at 6 mo training Difference in mean PCS: -2.6, *P*=.004 No significant difference in mean MCS
Jolly et al. 2007^55^ (2.7.2 Mode of service delivery)	15	Cardiac patients	Outpatient Center-based rehabilitation (n=236) Mean PCS ± SD: 42.6 ± 10.8 Mean MCS ± SD: 49.2 ± 10.1	Home-based rehabilitation (n=239) Mean PCS ± SD: 42.3 ± 10.9 Mean MCS ± SD: 50.3 ± 9.6	SF-12 at 12 mo follow-up Difference in mean PCS: 0.3† Difference in mean MCS: -1.1†
Maddison et al. 2019^56^ (2.7.2 Mode of service delivery)	15	Coronary disease	Outpatient rehabilitation (n=69) Mean ± SD: 0.89 ± 0.13	Home-based rehabilitation, tele-based (n=65) Mean ± SD: 0.92 ± 0.09	EQ-5D Index at 6 mo follow-up Difference in mean: -0.03, (-0.06 to 0.01)
Varnfield et al. 2014^57^ (2.7.2 Mode of service delivery)	15	Postmyocardial infarction	Outpatient rehabilitation Center based (n=38) Mean (95% CI): 0.82 (0.7-0.9)	Home-based rehabilitation Outreach smartphone (n=23) Mean (95% CI): 0.92 (0.9-1.0)	EQ-5D Index at 6 wk follow-up Difference in mean: -0.08 (-0.1 to -0.02) *P*=.01
Comans et al. 2010^58^ (2.7.2 Mode of service delivery)	12	Elderly	Community rehabilitation Center based (n=35) Mean ± SD: 0.78 ± 0.18	Community rehabilitation, home-based (n=41) Mean ± SD: 0.63 ± 0.31	EQ-5D index at 6 mo follow-up Difference in mean: 0.12 *P*=.11
Hwang et al. 2017^59^ (2.7.2 Mode of service delivery)	15	Chronic heart failure	Outpatient rehabilitation (n=26) Mean ± SD: 0.74 ± 0.25	Home-based rehabilitation, Tele-based (n=23) Mean ± SD: 0.73 ± 0.22	EQ-5D index at 6 mo follow-up Difference in mean: -0.06 (-0.16 to 0.01)
Lincoln et al. 2004^60^ (2.7.2 Mode of service delivery, 2.7.1 Levels of care)	12	Stroke	Day hospital (n=100) Median (IQT): 55 (40-72)	Home-based rehabilitation (n=88) Median (IQT): 52 (41-78)	EQ-5D (global) at 6 mo follow-up Difference in median: 3†, *P*=.75
Roderick et al. 2001^61^ (2.7.2 Mode of service delivery, 2.7.1 Levels of care)	13	Stroke	Day hospital rehabilitation (n=58) Median (95% CI) PCS: 32.7 (26.8-39.2) MCS: 57.1 (60.6-63.0)	Home-based rehabilitation (n=54) Median (95% CI) PCS: 35.2 (26.5-43.7) MCS: 57.4 (49.9-62.9)	SF-36 at 6 mo follow-up Difference in median PCS:-2.5†, *P*=.22 Difference in median MCS: -0.3†, *P*=.99
Crotty et al. 2008^62^ (2.7.2 Mode of service delivery)	14	Mixed	Day hospital rehabilitation (n=108) Mean PCS ± SD: 42.6 ± 10.2 Mean MCS ± SD: 47.3 ± 12.2	Home-based rehabilitation (n=114) Mean PCS ± SD: 42.7 ± 10 Mean MCS ± SD: 46.7 ± 12.4	SF-36 at 3 mo follow-up Difference in PCS median: -0.1† Difference in MCS median: 0.6†
Evans and Hendricks 2001^63^ (2.7.2 Mode of service delivery, 2.7.1.Levels of care)	10	Mixed disabilities	Home-based rehabilitation, outreach (n=90) Mean PCS ± SD: 100.3 ± 20.0	Community-based rehabilitation, as usual (n=90) Mean PCS ± SD: 100.3 ± 20.6	SF-36 at 12 mo follow-up Difference in mean: 0†, NS
Vasilopoulou et al. 2017^64^ (2.7.2 Mode of service delivery, 2.7.1 Levels of care)	13	COPD	Outpatient rehabilitation (n=50) Mean ± SD: 1.3 ± 0.9	Home-based rehabilitation, Tele-based (n=47), TAU (n=50) Tele: Mean ± SD: 0.6 ± 1.0 TAU: Mean ± SD: 3.1 ± 0.8	Medical research council dysponea scale (QoL) at 14 mo follow-up All groups significantly improved from baseline
**Functional Outcome**					
Patti et al. 2003^25^ (2.7.2 Mode of service delivery, 2.7.1 Levels of care)	14	MS	Outpatient rehabilitation (n=58) Mean ± SD: 103.0 ± 14.3 Mean change ± SD): 10.2 ± 11.8	Home-based exercises (n=53) Mean ± SD: 93.7 ± 16.4 Mean change ± SD: 0.0 ± 0.7	Total FIM at 3 mo follow-up Difference in total mean FIM: 9.3†, *P*<.001 Difference in mean change: 10.2†, *P*<.001
Powell et al. 2002^65^ (2.7.2 Mode of service delivery, 2.7.1 Levels of care)	14	TBI	Outpatient rehabilitation Outreach (n=48) Change in median (range) from baseline: 0.0 (-5 to 5), 35% show improvement	Information group, community-based (n=46) Change in median (range) from baseline 0.0 (-5 to 4), 20% show improvement	Barthel Index (range) at 6 mo follow-up Difference in improvement: 15%†
Burch et al. 1999^66^ (2.7.2. Mode of service delivery, 2.7.1 Levels of care)	13	Elderly	Day hospital rehabilitation (n=34) Change in mean (95% CI) from baseline: 1.5 (0.66-2.34)	Outpatient day center rehabilitation (n=38) Change in mean (95% CI) from baseline: 1.5 (0.53-2.47)	Barthel Index at 3 mo follow-up Difference in mean (95% CI): 0.0 (-1.28 to 1.28)
Bjorkdahl et al. 2006^67^ (2.7.2 Mode of service delivery)	14	Stroke	Day hospital rehabilitation (n=29) Mean motor logits ± SD: 2.99 ± 1.76 Mean cognitive logits ± SD: 3.29 ± 1.50	Home-based rehabilitation Patient centered (n=29) Mean motor logits ± SD: 3.14 ± 2.07) Mean cognitive logits ± SD: 2.68 ± 1.67	FIM at 12 mo follow-up Difference in mean logits: Motor: -0.15† Cognitive: 0.61†
Gladman et al. 1993^68^ (2.7.2 Mode of service delivery, 2.7.1 Levels of care)	12	Stroke	Outpatient rehabilitation (n=148) Overall Median (IQT):18 (15-20) Stroke unit only: 18 (15-19)	Home-based rehabilitation (n=134) Overall Median (IQT):17 (14-19) Stroke unite only: 16 (15-18)	Barthel Index at 6 mo follow-up Difference in median (95% CI): 1† Stroke unit only: 2.0†
Winter et al. 2016^26^ (2.7.2 Mode of service delivery)	15	TBI	Outpatient rehabilitation (n=35) Mean ± SD: 3.03 ± 0.64	Home-based rehabilitation Patient centered (n =36) Mean ± SD: 2.56 ± 0.79	Targeted outcome at 4 mo follow-up Patient centered HR group manages most difficult problems better, *P*=.02
Borg et al. 2018^24^ (2.7.2 Mode of service delivery)	12	Persons with reduced hearing	Outpatient rehabilitation Center based, hearing aids (n=65)	Community-based rehabilitation, hearing aids (n=75)	IOI-HA at 6 wk follow-up Both approaches performed equally well on 5 out of the 7 items; center approach did better on 2.
Lopez-Liria et al. 2015^69^ (2.7.2 Mode of service delivery, 2.7.1.Level of care)	9	Knee prosthesis	Outpatient rehabilitation (n=39) Mean ± SD 99.10 ± 6.65	Home-based rehabilitation (n=32) Mean ± SD: 97.19 ± 4.0	Barthel Index after intervention Difference in mean: 1.91†, NS Outpatient rehabilitation had better knee extension and muscle strength
Horton et al. 2018^70^ (2.7.2 Mode of service delivery)	13	COPD	Outpatient rehabilitation (n=83) Mean ± SD: 3.38 ± 1.20	Home-based rehabilitation Outreach (n=79) Mean ± SD: 3.15 ± 1.22	Chronic respiratory questionnaire at 7 wk after randomization Difference in mean: 0.23†, NS
Holland et al. 2017^71^ (2.7.2 Mode of service delivery)	15	COPD	Outpatient rehabilitation (n=76) Mean change from baseline (SD): 0.14 (-16.34 to 17.15)	Home-based rehabilitation Outreach (n=72) Mean change from baseline (SD): -4.74 (-21.94 to 12.47)	6-min walk test at 12 mo follow-up Difference in mean change: -4.6†, NS
Maltais et al. 2008^72^ (2.7.2 Mode of service delivery)	13	COPD	Outpatient rehabilitation (n=109) Mean change from baseline (SD): -5 (-17 to 7)	Home-based rehabilitation Outreach (n=107) Mean change from baseline (SD): 0 (-13 to 12)	6-min walk test at 12 mo follow-up Difference in mean change: 5†
Mendes de Oliveira et al. 2010^73^ (2.7.2 Mode of service delivery)	11	COPD	Outpatient rehabilitation (n=23) Significant improvement from baseline	Home-based rehabilitation (n=33), Control group (n=29) Significant improvement from baseline; no change in control	6-min walk test at 12 wk follow-up No significant group difference in improvement, *P*=.44

NOTE. Target group, quality score according to Cicerone 2009, brief mapping of the intervention arms with number of randomized subjects, and the outcome (generic functioning or quality of life measurement reported when multiple outcomes). Group difference, with effect size and statistical level reported when possible.

Abbreviations: COPD, chronic obstructive pulmonary disease; EQ-5D,; HR,; IOI-HA, International Outcome Inventory for Hearing Aids; MCS, Mental Component Summary; MS, multiple sclerosis; NS, not different statistically; PCS, Physical Component Summary; QoL,; SF-12,; TAU, treatment as usual; TBI, traumatic brain injury.

⁎ Information was partially reported, for example, the outcomes were reported graphicly only or within/between group difference were reported only, and so on.

† Outcome difference was calculated by current study descriptively (for example, 81 Jolly et al., Difference in mean PCS=42.6-42.3=0.3).

### Integration of Care (Category 2.8 in ICSO-R 2.0)

Six studies involving a total of 1792 patients that evaluated Integration of Care (delivery of rehabilitation in conjunction with other health services timely, comprehensive, and well coordinated according to the users’ needs) were identified. These studies were very different regarding target groups as well as the nature of the interventions, yet none of the studies documented significant differences regarding functional outcome or length of stay in relation to this factor (see table 5).

**Table 5:** Differences in Integration of Care (ICSO-R 2.0 category 2.8).

**Studies**	**Quality Rating** (Mean)	**Target Group** (Group Size)	**Intervention 1**	**Intervention(s) 2/3**	**Outcome** Intervention 1 vs Intervention 2/3
**2.8 Integration of care (co-variating categories)**					
Schaldach 1997^74^	10	Lower extremity Amputation	Inpatient rehabilitation, clinical pathway (n=46) Mean 8.0 ± SD 4.2 d	Inpatient rehabilitation consultation (n=34) Mean 12.8 ± SD 8.0 d Usual care (n=104) Mean 13.1 ± SD 7.2 d	Length of stay Difference in mean; Pathway vs consultation -4.8* Pathway vs usual care -5.2* No significant differences
Chan et al. 2014^75^	14	Stroke	Inpatient rehabilitation, integrated stroke unit (n=20) Mean 109.5 ± SD 21.7	Inpatient rehabilitation, separate stroke unit (n=21) Mean 104.4 ± SD 27.9	FIM 3 mo after discharge Difference in mean 5.1* No significant group difference
Gomez et al. 2017^76^ (2.10 Aspects of time)	11	Burn survivors	Inpatient rehabilitation, early integrated (n=78) Mean change 24.8 (12.3)	Inpatient later rehabilitation (n=60) Mean change 24.0 (14.7)	FIM Difference in mean change 0.8* No significant group difference
Wolfe et al. 2000^77^ (2.10 Time and intensity, 2.11.1 Professions and competencies)	12	Stroke	Home-based rehabilitation team (n=23) Median 18 (Range 8-20)	Home-based usual community care (n=20) Median 20 (Range 16-20)	Barthel, 12 mo Difference in median 2 No significant group difference
Indredavik et al. 2000^78^ (2.7.2. Mode of service delivery)	13	Stroke	Home-based rehabilitation, outreach with community collaboration (n=120) 65.0%	Community rehabilitation, usual care (n=120) 59.1%	mRankin, % ≤2, 6 mo, Difference 5.9% *P*=.017*
Attend collaborative group, 2017^79^	15	Stroke	Home-based, family-led rehabilitation (n=623) 47%	Home-based, usual care rehabilitation (n=627) 47%	mRankin ≥3, 6 mo Difference 0%* No significant group difference

NOTE. Target group, quality score according to Cicerone 2009, brief mapping of the intervention arms with number of randomized subjects, and the outcome (generic functioning or quality of life measurement reported when multiple outcomes). Group difference, with effect size and statistical level reported when possible.

⁎ Outcome difference was calculated by current study descriptively.

#### Patient Centeredness (Category 2.9 in ICSO-R 2.0)

Four studies with 1037 participants evaluated Patient Centeredness approaches (rehabilitation tailored to the person's needs and provided in partnership with them, their families, and communities). Only 1 of the studies with a total of 70 included patients favored patient-centered approaches (see table 6).

**Table 6:** Differences in patient centeredness (ICSO-R 2.0 category 2.9)

**Included studies**	**Quality Rating**(Mean)	**Target Group**	**Content** Intervention 1	**Content** Intervention 2	**Outcome**
**2.9 Patient Centeredness (co-variating categories)**					
Dambi and Jelsma 2014^80^ (2.7.2 Mode of service delivery*)*	8	CP	Outpatient rehabilitation (n=26) Mean 44.9 ± SD 19.8	Home-based rehabilitation, patient centered, outreach (n=20) Mean 43.5 ± SD 9.0	Gross Motor Function Measurement, 3-mo discharge Difference in mean 1.4* No significant group difference
Lewin et al. 2014^81^	11	Elderly	Home-based goal-oriented rehabilitation (n=375) AU$19.89	Home-based usual care plan (n=375) AU$22.76	Cost, 2-y aggregated Difference $3.87 No significant group difference
Vahedian-Azimi et al. 2016^82^	14	Cardiac patients	Home-based rehabilitation, family centered (n=35) Mean PCS 85.21 ± SD 4.65 Mean MCS 83.86 ± SD 3.81	Home-based rehabilitation, usual care (n=35) Mean PCS 23.01 ± SD 4.87 Mean MCS 20.44 ± SD 5.48	SF-36, 6 mo Difference in mean PCS 62.20*P<.0001 Difference in mean MCS 63.42*P<.0001
Gitlin et al. 2001^83^ (2.11.1 Professions, competencies)	10	Dementia	Home-based occupational therapist support to caregivers (n=93) Mean 3.24 ± SD 1.59	Usual care (n=78) Mean 3.57 ± SD 1.58	Caregiver reported patient dependency ADL (FIM modified), 3 mo Difference in mean -0.33* No significant group difference

NOTE. Target group, quality score according to Cicerone 2009, brief mapping of the intervention arms with number of randomized subjects, and the outcome (generic functioning or quality of life measurement reported when multiple outcomes). Group difference, with effect size and statistical level reported when possible.

Abbreviation: CP, cerebral palsy; MCS, Mental Component Summary; PCS, Physical Component Summary.

⁎ Outcome difference was calculated by current study descriptively.

### Aspects of Time and Intensity (Category 2.10 in ICSO-R 2.0)

In a total of 14 studies with 3037 patients, the differences in Time and Intensity were targeted. As illustrated in table 7, these 2 factors seem to matter in some cases. Yet, the variability of the target group, outcomes, and interventions prohibit more general conclusions.

**Table 7:** Differences in Aspects of Time and Intensity (ICSO-R 2.0 category 2.10)

Included studies	**Quality Rating** (Mean)	**Target Group**	**Content Intervention 1**	**Content Intervention 2**	**Outcome**
**2.10 Aspects of time and intensity (co-variating categories)**					
Peiris et al. 2012^37^	15	Orthopedic	Inpatient rehabilitation 6 d/wk (n=51) Mean 723 ± SD 674	Inpatient rehabilitation 5 d/wk (n=54) Mean 461 ± SD 583	Steps/d during rehabilitation Difference 262,*P=.04
Peiris et al. 2013^84^	15	Mixed	Inpatient rehabilitation 6 d/wk (n=496) Mean 284 ± SD 57	Inpatient rehabilitation 5 d/wk (n=500) Mean 266 ± SD 53	FIM, 12 mo Difference in mean 18* No significant group difference
Freyssin et al. 2012^85^	10	Cardiac failure	Inpatient interval training (n=12) Mean 475 ± SD 52 m	Inpatient continuous training (n=14) Mean 451 ± SD 72 m	6-min walk test, 8 wk Difference in mean 24* No significant group difference
Slade et al. 2002^86^ (2.10 Aspects of time and intensity)	14	Acquired brain injury	Inpatient rehabilitation, 67% larger amount (n=80) Median change 20 (IQR 9-32)	Inpatient rehabilitation (n=80) Median change 14 (IQR 2-28)	Barthel Index, discharge Difference in median change 6* No significant group difference
Bakheit et al. 2007^87^	14	Aphasic stroke	Inpatient rehabilitation intensive speech therapy (n=51) Mean 70.3 ± SD 26.9	†Inpatient rehabilitation, usual care (n=46) Mean 58.1 ± SD 33.7	Western Aphasia Battery, 12 wk Difference mean change 4.4* No significant group difference
MacPhee et al. 2004^88^	12	Wheelchair users	Inpatient rehabilitation including wheelchair training (n=18)	Inpatient rehabilitation (n=26)	Psychosocial impact of assistive device scale Difference in mean change 12.2*P<.001
Shiel et al. 2001^89^	15	TBI	Inpatient intensive rehabilitation (n=24) Mean difference of 7 subscales reported	Inpatient rehabilitation, usual care (n=27) Mean difference of 7 subscales reported	7 subscales of FIM/FAM at discharge Difference in mean change all 7 subscales, *P*<.01
Ruff et al. 1999^90^	12	Stroke	Inpatient rehabilitation 7 d/wk (n=56) Improvement subscales bladder, ambulation, dressing, problem-solving reported	Inpatient rehabilitation 6 d/wk (n=57) Improvement subscales bladder, ambulation, dressing, problem-solving reported	FIM subscales, discharge No significant group differences in any subscale
Roman et al. 2013^91^	12	COPD (n=26+22+23)	Outpatient rehabilitation 9 mo maintenance (n=26) Mean and CI for subscales of CRQ reported	Outpatient rehabilitation, 3 mo maintenance (n=22) Treatment as usual (n=23) Mean and CI for subscales of CRQ reported	CRQ, 12 mo No significant group difference (numerous scales)
Khan et al. 2011^92^ (2.9 Patient Centeredness)	14	Guillan Barre	Inpatient rehabilitation, high intensity (n=40) Median change 4 (IQR 0-7)	Inpatient rehabilitation, usual care (n=39) Median change 0 (IQR -3 to 5)	FIM, 12 mo Difference median change 4 *P=.003
Bondestam et al. 1995^93^ (2.11.1 Profession and Competencies, 2.10 Time and Intensity).	10	Myocardial infarct	Community-based rehabilitation (n=91) 32%	Control group (n=99) 47%	Rehospitalization % 12 mo Difference 15%, *P*=.05* (rehospitalization measured in days community-based rehabilitation < control)
Gräsel et al. 2006^94^ (2.9 Patient Centeredness, 2.8 Integration of Care)	10	Stroke	Inpatient rehabilitation, intensive transition care (n=36)	Inpatient rehabilitation as usual (n=35)	Dependency 31 mo Group difference data not reported; but intensive transition care reported as a significant predictor of living at home after 31 mo (regression analysis)
Morreale et al. 2016^95^	12	Stroke	Inpatient early rehabilitation/mobilization (n=220 divided in neuromuscular n=110 and cognitive n=110 interventions)	Inpatient later rehabilitation (n=120 divided in neuromuscular n=60 and cognitive n=60 interventions)	Barthel, 12 mo Difference in mean change 9 in neuromuscular subgroup*P<.02 Difference in mean change 4 in cognitive subgroup*P<.02
Bouman et al. 2017^96^ (2.8 Integration of Care)	12	Multitrauma	Inpatient rehabilitation, fast track integrated (n=65) Mean 119.0 (SE 1.34)	Inpatient rehabilitation, integrated (n=67) Mean 120.6 (SE 1.26)	FIM 12 mo Difference in mean -1.6* No significant group difference

NOTE. Target group, quality score according to Cicerone 2009, brief mapping of the intervention arms with number of randomized subjects, and the outcome (generic functioning or quality of life measurement reported when multiple outcomes). Group difference, with effect size and statistical level reported when possible.

Abbreviations: CQR, chronic respiratory questionnaire; IQR, .

⁎ Outcome difference was calculated by current study descriptively.

† Data from additional non-randomized reference group was not included.

### Multiple categories

Finally, in 12 studies, multiple aspects of the Service Provider and Delivery dimensions were clearly targeted but covaried in the intervention arms, which impedes the drawing of any conclusion regarding their influence on the outcomes or renders the categorization in ICSO-R 2.0 challenging (see supplemental S2).

## Discussion

This is the first systematic review to comprehensively screen and synthesize the effects of rehabilitation Service Provider and Delivery categories as described in ICSO-R 2.0 on patients’ functional outcome in intervention studies.

Building evidence on the effects of the organizational factors in rehabilitation services is a major challenge.^27^ The present review clearly underscores the challenges not only in large variations in the reporting of service-related factors but also in the presentation of the outcomes. This variability represents an obstacle for the aggregation of data and evidence across studies.^28^ There are several reasons for this variability. First of all, the rehabilitation services embrace persons with a large variety of medical diagnoses. In addition, the nature of the functional problems, the level of disability as well as age and phase of disease are important factors for the organization and content of the rehabilitation.^29^ To develop evidence-based and effective rehabilitation services, there is an apparent need for comparing and aggregating data across studies through reviews and meta-analysis. Among the more than 10,000 identified articles in the present review, only 80 studies clearly described organizational differences in the intervention arms. In another 12 studies, covariation between several organizational factors was too large to support further analysis. The lesson from the development of ICF is relevant in this context, with a large increase in scientific evidence in the field of functioning brought about by a common language.^30^

Several theories and models have been developed for the evaluation of quality in the rehabilitation services,^31^ but to our knowledge no common language or classification system has been established beyond the proposed ICSO-R. ICSO-R thus represents an increased possibility to move forward in the development and implementation of this classification system in clinical rehabilitation in a similar way as experienced with the ICF. Specifications of individual interventions may be combined with standardization of the service aspects to provide a more complete characterization of rehabilitation provision.^32^

We would also advocate further improvement in outcome reporting. Although most of the studies reported on functioning or quality of life in addition to more specific measurements, the differences in between-group changes were often difficult to extract and raw data difficult to access.

The most frequently reported and analyzed organizational factor in the present review was Setting, in particular the subcategory Mode of Service Delivery. In more than half of the included studies, the intervention arms varied according to this category. In addition, Mode of Service Delivery seemed to have effects on functional outcome. Yet, caution is needed because covariation of other organizational factors may have been underestimated. In particular, the resources and professions and team members are very likely to covary across settings.

We had to divide the analysis in 3 groups: comparisons in inpatient setting, between in- and outpatient settings, and between different outpatient settings. This clearly demonstrates the necessity of developing well-defined value sets for the categories and subcategories in ICSO-R.

The present results are in support of integrated stroke rehabilitation compared with ward-based care. The studies were, as expected, old because stroke units being part of the national recommendations and treatment in stroke units is no longer considered ethical to omit.^33^ With the exception of the study by Mutwalli et al,^18^ the present results did not favor in- vs outpatient settings, nor particular outpatients modes of service delivery. However, when comparing settings across hospitals and communities, human and other resources as well as team, competence, and provider also covary. Furthermore, these factors may also covary with the content of the interventions or the study population in the different interventions. In the included RCTs the differences in study population characteristics were generally controlled for in the analysis. Improved standardized reporting will be the first step to enable multivariate statistical analyses to control for multiple differences in the intervention arms and their effect on the outcomes. Yet, the fact that this review indicates a lack of differences between rehabilitation services provided in outpatient, home-based, or community settings, implies, especially in the view of the current pandemic situation, that home-based training might be an option for future rehabilitation. This shows that the ICSO-R system offers researchers the opportunity to provide other researchers with information that goes beyond their own study goals and can improve rehabilitation services in the long-term. Hence, we would clearly advice the implementation of reporting requirements for settings of service delivery.

For Integration of Care and Patient Centeredness, indifferent results across the interventions were found. These are clearly important quality aspects of rehabilitation.^34^ Yet, identifying the effect of these factors in RCTs may be challenging, and summarizing such results in meta-analysis may not be meaningful. Further exploration of which features of Patient Centeredness are the most important in different situations with mixed-methods approaches could be a way to move forward.^35^

We need more knowledge of the effects of Aspects Time and Intensity in the rehabilitation services. Negative findings are as important as positive ones. Time and Intensity may not always be easily assessed as the effects may be more closely related to the intervention adherence more than what is being prescribed.^36^ Studies such as Peiris et al^37^ comparing Time and Intensity, that is, rehabilitation provided 6 vs 5 days a week need to be controlled for other confounding factors. In contrast, comparing settings across hospitals and communities generally implies variations in human and other resources as well as team, competence, and provider differences. The Template for Intervention Description and Replication provides important recommendations for improved reporting of interventions.^38^ Yet, more rigorous categorization and classification of the time and intensity aspects along with the other organizational factors are needed to apprehend more fully the influence of these factors.^39^

### Study limitations

There are main limitations in the present review. Several included studies were conducted before 2010, and hopefully reviews of more current RCT reports will benefit from the increased adherence to the Consolidated Standards of Reporting Trials guidelines in clinical trials. The current review includes only RCTs, which may limit the generalizability of the results to studies with longitudinal observational design. We may have lost many important studies in the screening process by reading only the abstracts to identify studies that met the inclusion criteria because of the possible omission of details regarding service provision and delivery in the abstracts or methods. Furthermore, we did not include diagnostic terms for all possible conditions in need of rehabilitation in the search. The standardized evaluation manuals and value sets of ICSO-R 2.0 are not yet developed; thus, the data extraction and classification of rehabilitation services may be biased by the outlook and experience of the authors. Still, the largest limitation is that the interventions delivered are likely to vary among different service and delivery aspects, making it difficult to determine the extent to which differences in outcome are driven by service delivery characteristics vs the interventions themselves.

## Conclusions

Organization of rehabilitation services (mesolevel) may affect functional outcome (microlevel). The present review has shown that settings and particularly the way the services were delivered to the users influenced functional outcome. Hence, it should be compulsory to include a standardized reporting of the organizational aspects of settings in clinical trials. We would also advise that the description of organizational factors in rehabilitation interventions should be further standardized so that our knowledge of factors associated with rehabilitation service organization can be accumulated systematically, which in turn can lead to more effective rehabilitation service delivery.

## Appendix Supplementary materials

Image, application 1

## Footnotes

Supplementary material associated with this article can be found, in the online version, at doi:10.1016/j.arrct.2022.100197.
