# Supplementary material for: Organization of Rehabilitation Services in Randomized Controlled Trials: Which Factors Influence Functional Outcome? A Systematic Review
Source: Arch Rehabil Res Clin Transl. 2022 Apr 13;4(2):100197. doi: 10.1016/j.arrct.2022.100197 (PMC9214333; doi:10.1016/j.arrct.2022.100197)
Supplement: Supplementary file 1 [file mmc1.docx]

**Supplement 1**. Search strategy

1 rehabilitation/ or cardiac rehabilitation/ or neurological rehabilitation/ or stroke rehabilitation/ or "rehabilitation of speech and language disorders"/ or rehabilitation, vocational/ or telerehabilitation/ or psychiatric rehabilitation/ or Rehabilitation Nursing/ or (rehabilit* or habilit* or prehabilit* or telerehabilitation).tw,kf. or rehabilitation.fs.

2 "Delivery of Health Care"/ or "Delivery of Health Care, Integrated"/ or (((deliver* or provid* or provision or integrat*) adj3 service*) or (deliver* adj2 home) or (integrated adj2 care) or (integrated adj2 program*)).tw,kf.

3 Health Services/ or Occupational Therapy/ or adolescent health services/ or community health services/ or child health services/ or community mental health services/ or home care services/ or home care services, hospital-based/ or ((home adj2 service*) or (rehabilitation adj2 (service* or program*)) or ((therapy or fracture) adj program*) or (health adj2 service*) or very early rehabilitation or early initiated or clinical service* or physiotherapy or physical therapy or occupational therapy).tw,kf.

4 or/2-3

5 1 and 4

6 health services administration/ or "organization and administration"/ or clinical governance/ or decision making, organizational/ or health facility administration/ or exp hospital administration/ or (organisat* or organizat* or administrat* or governance).tw,kf.

7 Rehabilitation centers/ or Hospitals, Rehabilitation/ or home health nursing/ or home nursing/ or ((hospital* adj3 (unit* or department*)) or (hospital adj2 based) or (community adj2 (based or health or nurs*)) or stroke center* or stroke centre* or rehabilitation center* or rehabilitation centre*).tw,kf.

8 organizations/ or health planning organizations/ or health care coalitions/ or health planning councils/ or "state health planning and development agencies"/ or health systems agencies/ or home care agencies/ or exp organizations, nonprofit/ or public-private sector partnerships/ or (private or public or nonpublic or nonprofit* or non profit* or for profit* or forprofit* or agenc*).tw,kf.

9 Patient-Centered Care/ or (patient cent* care or person cent* care).tw,kf.

10 residential facilities/ or assisted living facilities/ or homes for the aged/ or nursing homes/ or intermediate care facilities/ or skilled nursing facilities/ or (home based or nursing home* or home nurs* or home care or home health care or facility or facilities or intermediate care).tw,kf.

11 economics, hospital/ or hospital charges/ or hospital costs/ or financing, organized/ or financing, government/ or public assistance/

12 "health care economics and organizations"/ or economics/ or financial management/ or financial management, hospital/ or financial support/ or healthcare financing/ or financing, organized/ or financing, government/ or (economic* or fund* or financ* or charit*).tw,kf.

13 or/6-12

14 5 and 13

15 (randomis* or randomiz* or randomly or trial or effect? or controlled or control group* or quasiexperiment* or quasi experiment* or rct).ti,ab.

16 14 and 15

17 limit 14 to (clinical trial, all or clinical trial, phase i or clinical trial, phase ii or clinical trial, phase iii or clinical trial, phase iv or clinical trial or controlled clinical trial or randomized controlled trial)

18 16 or 17

19 limit 18 to "reviews (best balance of sensitivity and specificity)"

20 18 not 19

21 review.ti.

22 20 not 21

**Supplement 2.** Items rated regarding study quality

Eligibility Criteria Specified

Method of Randomization Described

Treatment Allocation Concealed

Similarity of Baseline Characteristics

Treatment and Control Interventions Described

Cointerventions Avoided or Equivalent

Outcome Measurement Blinded

Outcome Measures Relevant

Withdrawal and Dropout Rates Described

Short-Term Outcomes Measured

Long-Term Outcomes Measured

Timing of Outcome Measures Equivalent

Sample Size Described

ITT Analysis

Point Estimates and Variability Provided

Statistical Comparison of Treatment Effects

-

**Supplement 3.**

Studies with intervention arms revealing various differences in the rehabilitation service organization.

| **Studies** | **Target**  **Group** | **Content**  Intervention 1 | **Content**  Intervention 2 | **Outcome** |
| --- | --- | --- | --- | --- |
| Cunliffe et al. 2004 (97)  1.1 Context, 2.5 Location, 2.7.2 Mode of service Delivery | Elderly | Early discharge and general practitioner and rehabilitation team (n=185) | In and outpatient rehabilitation (n=185) | Death and in institution |
| Finnerty, Keeping et al. 2001 (98)  2.10 Time and intensity , 2.7.2 Mode of service delivery | Pulmonary disease | Outpatient rehabilitation, outreach (n=36) | Outpatient routine rehabilitation (n=29) | St Georges Respiratory Questionnnaire  (6 min walk test) |
| Eagle et al. 1991 (99)  2.11,1 Profession and competencies, 2.11.2 Interaction approaches, 2.7.2 Mode of service delivery, 2.7 Facility | Geriatric patients | Day hospital rehabilitation, teambased (n=55) | Inpatient, outpatient or communitybased rehabilitation (n=58) | Barthel (and Rand) |
| Pozzilli, Brunetti et al. 2002 (100)  1.1 Context, 2.7.2 Mode of Service delivery, | MS | Home hospital rehabilitation  (n=133) | Routine hospital care- outpatient  (n=68) | SF-36 |
| Oshima, Sono et al. 2014 (101)  2.9 Patient centeredness, 2.11.1 Competencies and Professions | Disabilities | Individual placement and support (n=18) | Vocational rehabilitation  (n=19) | Working hours |
| Harding, Leggat et al. 2013 102Waiting list organization (2.4 Modes of referral ) | Musculo-skeletal conditions | Scheduled appointments  (outpatient) (n=308) | Waiting list based  Appointments (outpatient)  (n=175) | Time from referral to 1.appointment  (EQ-5D) |
| Haig, Nagy et al. 1995 (103)  2.11,1 Profession and competencies, 2.11.2 Interaction approaches,2.6 Facility | Complex disabilities | Team evaluation (outpatient)  (n=21) | Office evaluation physiatrist(n=19) | Barthel (Frenchday AI) |
| Allen, McIntyre et al. 2016 (104  Population characteristics (2.5.2 Location characteristic) | Stroke | Urban population  (n=502) | Rural population  (n=278) | FIM, health services |
| Widén Holmqvist, von Koch et al. 1998 (105)  7.2 Mode of service delivery, 2.7.1 Level of care | Stroke | Homebased rehabilitation, outreach early discharge  (n=41) | Rehabilitation usual care  (Hospital, Day, home) (n=40) | Barthel |
| Klingels, Feys et al. 2013 (106)  2.10 Time and Intensity, 2.11,1 Profession and competencies, 2.11.2 Interaction approaches | CP | Inpatient, Constraint  movement+ intensive  rehabilitation (n=25) | Inpatient Constraint movement  (n=26) | Assisting Hand Assessment |
| Buhagiar, Naylor et al. 2017 (107)  2.7.2 Mode of service delivery | Kne arthroplasthy | Inpatient rehabilitation + homebased/outpatient (n=81) | Homebased/outpatient rehabilitation (n=84)  Control (n=112) | 6-min walk test (EQ-5D) |
| Friedland and McColl 1992 (108)  2.9 Patient centeredness, 2.11.1 Professions and competencies | Stroke | Homebased rehabilitation social support (n=48) | Homebased rehabilitation, treatment as usual (n=40) | Social support  (General Health Questionnaire) |

**Supplemental references**

97. Cunliffe AL, Gladman JR, Husbands SL, Miller P, Dewey ME, Harwood RH. Sooner and healthier: a randomised controlled trial and interview study of an early discharge rehabilitation service for older people. Age Ageing 2004;33:246–52.

98. Finnerty JP, Keeping I, Bullough I, Jones J. The effectiveness of outpatient pulmonary rehabilitation in chronic lung disease: a randomized controlled trial. Chest 2001;119:1705–10.

99. Eagle DJ, Guyatt GH, Patterson C, Turpie I, Sackett B, Singer J. Effectiveness of a geriatric day hospital. CMAJ 1991;144:699– 704.

100. Pozzilli C, Brunetti M, Amicosante AM, et al. Home based management in multiple sclerosis: results of a randomised controlled trial. J Neurol Neurosurg Psychiatry 2002;73:250–5.

101. Oshima I, Sono T, Bond GR, Nishio M, Ito J. A randomized controlled trial of individual placement and support in Japan. Psychiatr Rehabil J 2014;37:137–43.

102. Harding K, Leggat S, Bowers B, Stafford M, Taylor N. Reducing waiting time for community rehabilitation services: a controlled before-and-after trial. Arch Phys Med Rehabil 2013;94:23–31.

103. Haig A, Nagy A, LeBreck D, Stein G. Outpatient planning for persons with physical disabilities: a randomized prospective trial of physiatrist alone versus a multidisciplinary team. Arch Phys Med Rehabil 1995;76:341–8.

104. Allen L, McIntyre A, Janzen S, et al. Community stroke rehabilitation: how do rural residents fare compared with their urban counterparts? Can J Neurol Sci 2016;43:98–104.

105. Widén Holmqvist L, von Koch L, Kostulas V, et al. A randomized controlled trial of rehabilitation at home after stroke in southwest Stockholm. Stroke 1998;29:591–7.

106. Klingels K, Feys H, Molenaers G, et al. Randomized trial of modified constraint-induced movement therapy with and without an intensive therapy program in children with unilateral cerebral palsy. Neurorehabil Neural Repair 2013;27:799–807.

107. Buhagiar MA, Naylor JM, Harris IA, et al. Effect of inpatient rehabilitation vs a monitored home-based program on mobility in patients with total knee arthroplasty: the HIHO randomized clinical trial. JAMA 2017;317:1037–46.

108. Friedland JF, McColl M. Social support intervention after stroke: results of a randomized trial. Arch Phys Med Rehabil 1992;73:573–81.
